# Re-evaluating the kinetics of ATP hydrolysis during initiation of DNA sliding by Type III restriction enzymes

**DOI:** 10.1093/nar/gkv1154

**Published:** 2015-11-03

**Authors:** Júlia Tóth, Jack Bollins, Mark D. Szczelkun

**Affiliations:** DNA–Protein Interactions Unit, School of Biochemistry, University of Bristol, Bristol BS8 1TD, UK

## Abstract

DNA cleavage by the Type III restriction enzymes requires long-range protein communication between recognition sites facilitated by thermally-driven 1D diffusion. This ‘DNA sliding’ is initiated by hydrolysis of multiple ATPs catalysed by a helicase-like domain. Two distinct ATPase phases were observed using short oligoduplex substrates; the rapid consumption of ∼10 ATPs coupled to a protein conformation switch followed by a slower phase, the duration of which was dictated by the rate of dissociation from the recognition site. Here, we show that the second ATPase phase is both variable and only observable when DNA ends are proximal to the recognition site. On DNA with sites more distant from the ends, a single ATPase phase coupled to the conformation switch was observed and subsequent site dissociation required little or no further ATP hydrolysis. The overall DNA dissociation kinetics (encompassing site release, DNA sliding and escape via a DNA end) were not influenced by the second phase. Although the data simplifies the ATP hydrolysis scheme for Type III restriction enzymes, questions remain as to why multiple ATPs are hydrolysed to prepare for DNA sliding.

## INTRODUCTION

The ATPase activity of helicases is significantly activated upon substrate binding (i.e. to single or double-stranded DNA or RNA, depending on the enzyme). The classical view is that all helicases can be defined as translocases where the ATPase activity is required to maintain repeated domain motions coupled to movement along the polynucleotide track (1). In turn this movement produces a functional outcome, such as duplex unwinding or remodelling of an adjacent nucleoprotein complex. The Type III restriction endonucleases (REs) such as the related enzymes EcoP15I and EcoPI illustrate an additional molecular switch role for Superfamily 2 helicase-like motors, where the ATPase activity is required to change protein conformation and initiate the long-range motion of the enzyme (2–9). There is no evidence that ATP hydrolysis is required for strand separation (i.e. classical helicase activity). ATP-driven dissociation of EcoP15I from a site followed a simple single exponential kinetics (4). In spite of that, the accompanying ATPase activity was surprisingly complex; multiple ATPs were hydrolysed in two kinetically distinct burst phases. Here we investigated further the Type III ATPase activity, and provide evidence for a simplified molecular switch model where only a single burst phase is necessary to drive the molecular switch.

**Figure 1.**
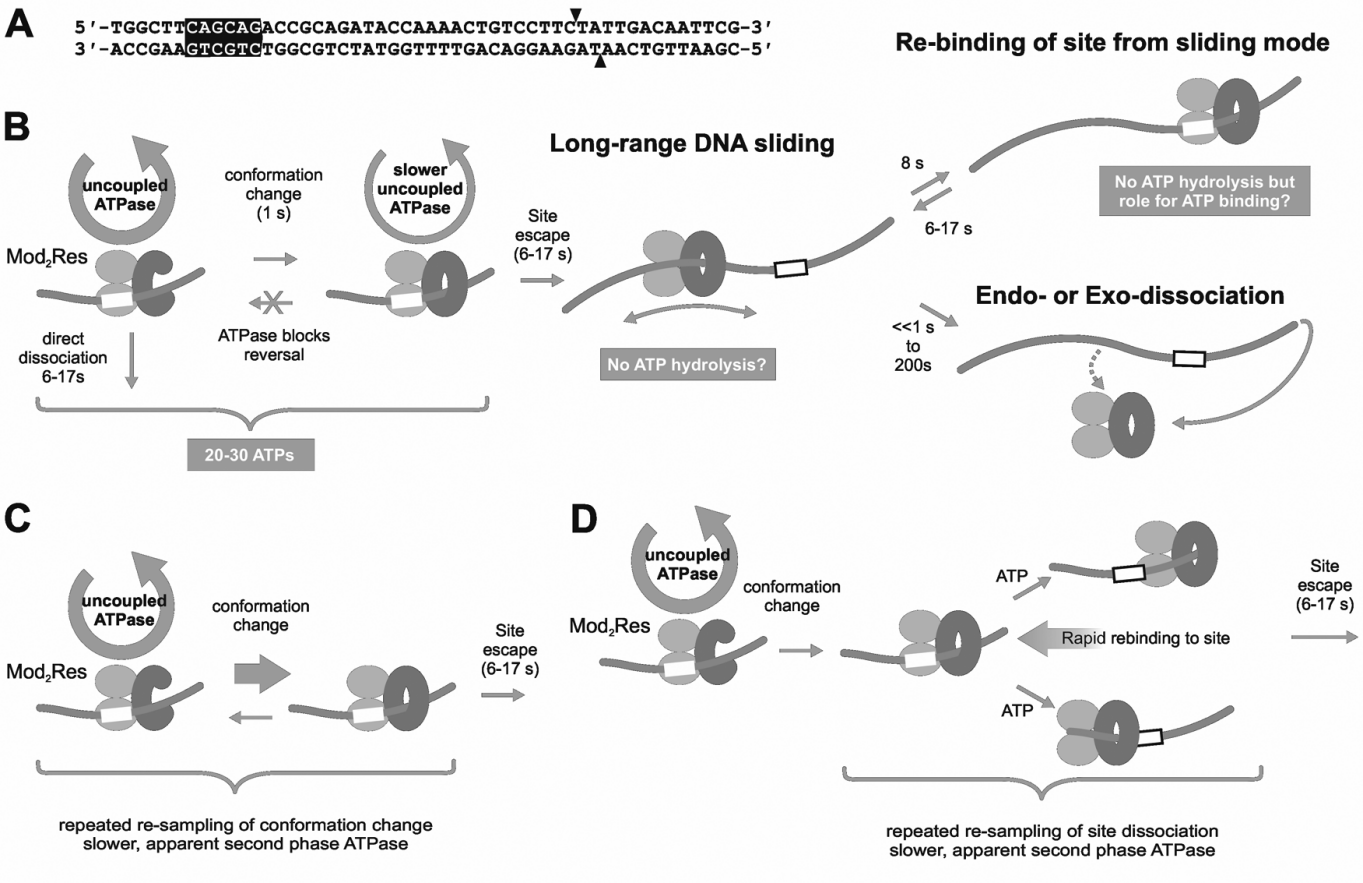
Models for ATPase-coupled conformation switching to a DNA sliding state by Type III restriction-modification enzymes. (**A**) EcoP15I specific oligoduplex substrate (6/38_P15I) used here, and previously (4). Arrowheads indicate the sites of cleavage. The nomenclature reflects the position of the recognition site in the sequence; e.g. the CAGCAG recognition sequence is 6 base pairs from the 5′ end and 38 base pairs from the 3′ end. (**B**) Dual phase ATPase cycles proposed previously based on single molecule and biochemical studies (3,4,9,20). Mod subunits are shown as light grey ellipsoids. Res subunits are shown as a dark grey cee-shape (prior to the switch) or a dark grey circle (post-switch sliding state). The recognition site is a white box. (**C**) Alternative model where reversal of the conformation switch leads to the observed second burst phase of ATP hydrolysis. (**D**) Alternative model where rebinding to the site following initiation of sliding leads to the observed second burst phase of ATP hydrolysis. See main text for more details.

In the absence of ATP, Type III REs bind tightly to their recognition sites (e.g. 5′-CAGCAG-3′ for EcoP15I or 5′-AGACC-3′ for EcoPI). Using a short oligoduplex substrate (Figure 1A), pre-bound by EcoP15I, we initiated an ATPase reaction by rapidly mixing with ATP and heparin (4). The heparin was included to trap any Type III enzymes that dissociated from the DNA. Two phases of ATP hydrolysis were observed: an initial rapid burst phase lasting ∼1 s during which ∼10 ATPs were consumed; and a second slower burst phase which decayed as a single exponential with a time constant of ∼8 s, during which >20 ATPs were consumed (Figure 1B). We were able to correlate the first ATP burst with a protein conformation switch as changes in EcoP15I tryptophan fluorescence occurred over a similar timescale (4). The exponential rate constant of the second burst phase matched closely the exponential rate constant for EcoP15I dissociation from a fluorescent version of the oligoduplex (reported as a change in anisotropy). By directly observing quantum dot-labelled EcoP15I on a ∼25 kilobase pair (kbp) DNA substrate using single-molecule fluorescence microscopy, we showed that ATP hydrolysis-driven release from the site led to one dimensional DNA diffusion (sliding) (Figure 1B). Sliding was both fast (∼16 × 10^6^ random single nucleotide steps per second) and long-lived (the lifetime before dissociation from internal sites—endo-dissociation—was ∼200 s). However, sliding of EcoP15I off the fluorescent oligoduplex via the DNA ends (exo-dissociation) would decrease the sliding lifetime to microseconds (3,5,9,10). Exo-dissociation was thus taken to be near instantaneous following release from the site. Hence, we correlated both the change in anisotropy and decay in the second ATPase burst phase as being the release from the site into the sliding state (4). Dissociation via a DNA end would only become rate-limiting with respect to the ATPase kinetics with site-to-end distances in excess of 12 kb.

We proposed that ATP binding and hydrolysis had two nucleoprotein remodelling roles (4): destabilizing the DNA-binding interface to allow release of the specific sequence and access to adjacent non-specific sites; and, changing the protein conformation to produce an undefined ‘DNA sliding’ state that can remain in contact with the DNA for tens of seconds whilst also allowing rapid and random transfer between adjacent non-specific sites driven by thermal motion. The change in conformation associated with the first ATPase burst could be one or other, or both, of these events. We suggested that the second ATPase burst phase was required to maintain a stressed conformation until the enzyme could release the site and start sliding (4,6). The different ATP hydrolysis rates in each observed burst phase were rationalized as different mechanochemical coupling efficiencies pre- and post-conformation switch. However, an alternative view is that following the conformation switch there is no necessity to consume any ATP to maintain the stressed state or to leave the site; i.e. only a single ATPase burst is absolutely necessary for DNA sliding. The observed second ATPase phase may instead arise because of alternative, hidden events that require additional rounds of ATP hydrolysis, including: rebinding of the site following initiation of sliding, which has been directly observed in the single molecule sliding assay (Figure 1D) (4); and/or reversal of the conformation change (Figure 1C). In both cases, the observed rate of the second phase would be slower than the first because only a fraction of enzymes would rebind and/or reverse.

To try to resolve the complexities of the Type III RE ATPase mechanism, we investigated whether the same, dual ATPase phases and conformational switch were observed with the related enzyme EcoPI on an oligoduplex substrate, or with EcoP15I using alternative, longer DNA substrates. To monitor dissociation from the longer DNA we developed a surface plasmon resonance (SPR) assay to follow DNA binding and dissociation by Type III REs. This assay also allowed us to expose the enzyme–DNA complex to short pulses of ATP to observe whether dissociation from the DNA required prolonged ATP hydrolysis. Our combined data is consistent with a simplified model where a single round of ATP hydrolysis is sufficient for the conformational switch and for dissociation from the site. Moreover, the data is consistent with a model where continued ATP hydrolysis is not required to maintain the sliding state but that ATP binding may be important in preventing irreversible re-binding to the recognition site.

## MATERIALS AND METHODS

### DNA and proteins

EcoP15I, EcoPI and AddA^H^B were expressed and purified as previously described (3,11). The following oligonucleotides (from MWG-Biotech AG) were annealed in a 1:1 ratio to produce 50 bp duplex substrates: 5′-TGGCTTCAGCAGACCGCAGATACCAAAACTGTCCTTCTATTGACAATTCG-3′ and 5′-CGAATTGTCAATAGAAGGACAGTTTTGGTATCTGCGGTCTGCTGAAGCCA-3′ for 6/38_EcoP15I; 5′-TGGCTTAGACCACCGCAGATACCAAAACTGTCCTTCTATTGACAATTCG-3′ and 5′-CGAATTGTCAATAGAAGGACAGTTTTGGTATCTGCGGTGGTCTAAGCCA-3′ for 6/38_EcoPI. To make pKA16.5, reverse PCR using primers 5′-GACGAAGGCTTGAGCGAGGG-3′ and 5′-CGGGTGATGCTGCCAACTTACTG-3′ was used to delete a section of DNA between the EcoP15I sites of pKA16 (4). The DNA for the SPR experiments (89/206Bio_P15I) was generated by PCR from pKA16.5 using the primers 5′-GGATGTGCTGCAAGGCGATTAAG-3′ and 5′-biotin-AAAATGACCCAGAGCGCTGCC-3′. For the end dependence experiments, the DNA was generated by PCR from pKA16.5 using the following primers: 5′-CACAGATGCGTAAGGAGAAAATACCGCATCAGGCGCC-3′ and 5′- AAAATGACCCAGAGCGCTGCC-3′ (for 206/206_P15I); 5′-TGGCTTCAGCAGACCGCAGATACCAAAACTGTCCTTCTATTGACAATTCG-3′ and 5′-AAAATGACCCAGAGCGCTGCC-3′ (for 6/206_P15I); 5′-CACAGATGCGTAAGGAGAAAATACCGCATCAGGCGCC-3′ and 5′-CGAATTGTCAATAGAAGGACAGTTTTGGTATCTGCGGTCTGCTGAAGCCA-3′ for (206/38_P15I). The nomenclature of the DNA substrates reflects the position of the recognition site in the sequence, e.g. for the 6/38_P15I substrate, the CAGCAG recognition sequence is 6 base pairs away from the 5′ end and 38 base pairs away from the 3′ end.

### ATPase assay

**Figure 2.**
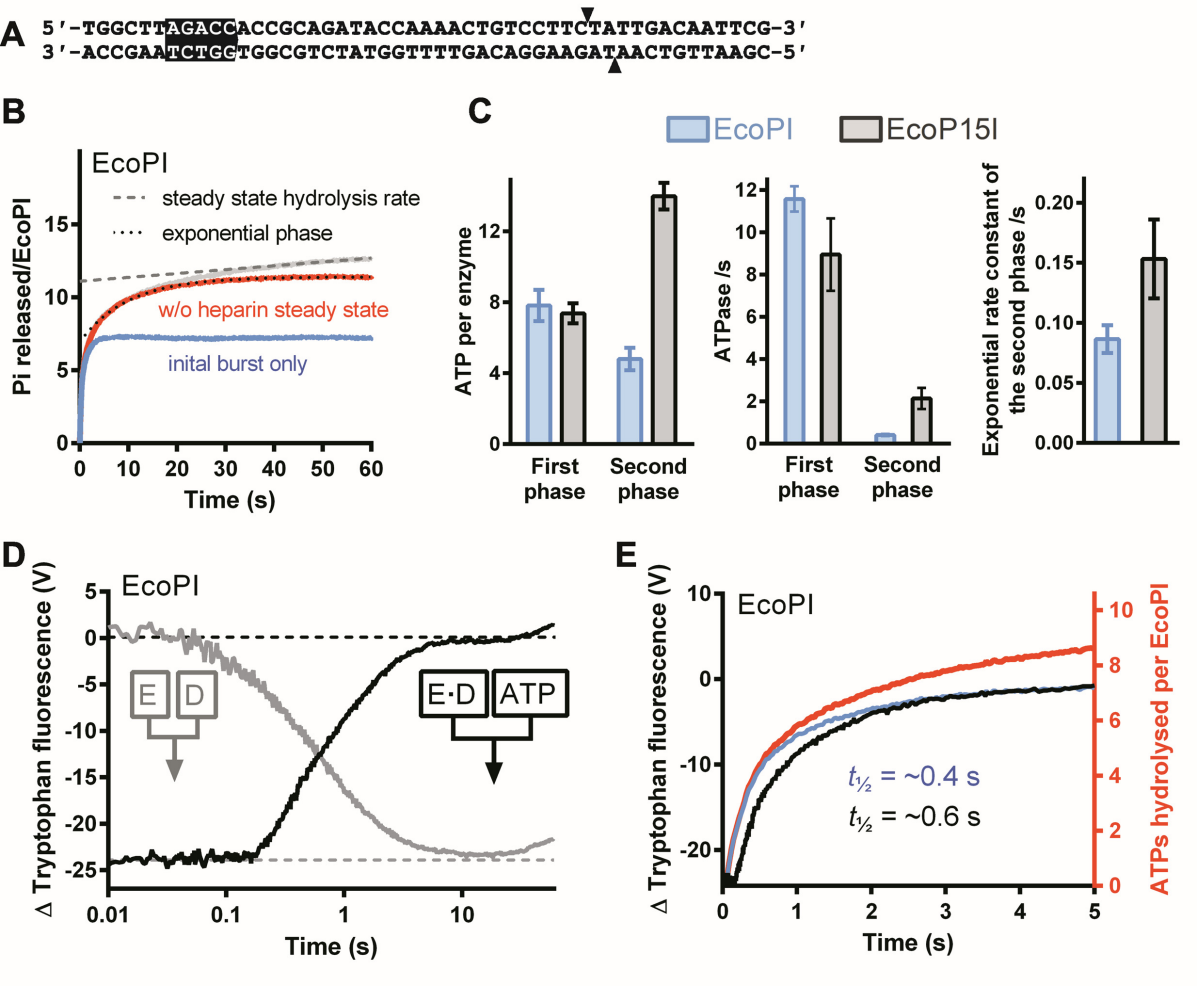
ATPase kinetics of EcoPI on a specific oligoduplex. (**A**) EcoPI specific oligoduplex substrate (6/38_PI) used here. Arrowheads indicate the sites of cleavage. (**B**) Preformed EcoPI–oligoduplex complex (25 nM) was rapidly mixed with 4 mM ATP and 200 μM heparin, and the release of phosphate measured (Materials and Methods). Rebinding to the DNA was prevented by the heparin trap. The data was deconvoluted as described in Materials and Methods (4) and shown in Supplementary Figure S1B–D. The grey trace represents the measured unprocessed data from which the steady state corrected trace (red line) is derived by subtracting a linear function (dashed line) fitted to the later stages of the grey trace. The corrected data (red) was fitted to Eq. (2) (dotted line). We can visualize the theoretical first burst phase (blue) by subtracting the exponential function from the corrected profile. (**C**) Kinetic constants determined for EcoPI on 6/38_PI and EcoP15I on 6/38_P15I. The number of ATPs consumed in the first and second bursts phases were obtained from the *A*_1_ and *A*_2_ values, respectively, from fits to Eq. (1). The first burst phase ATPase rate was obtained using a linear fit to the initial part of the burst (Supplementary Figure S1D). The ATPase rate of the second phase was calculated using Eq. (2). Averages and standard deviations were calculated from at least two repeats. (**D**) Measurement of EcoPI conformation change by tryptophan fluorescence measured in a stopped flow fluorimeter (Materials and Methods). The effect of DNA association was measured by mixing EcoPI with 6/38_PI (grey). The effect of DNA dissociation was measured by mixing pre-formed EcoPI–oligoduplex with ATP (black). Final reaction conditions were 1 μM DNA, 250 nM EcoPI, 4 mM ATP and 500 μM heparin. (**E**) Comparison of the complete (red) and burst (blue) ATPase profiles from panel B with the tryptophan fluorescence changes (black) from panel D.

**Figure 3.**
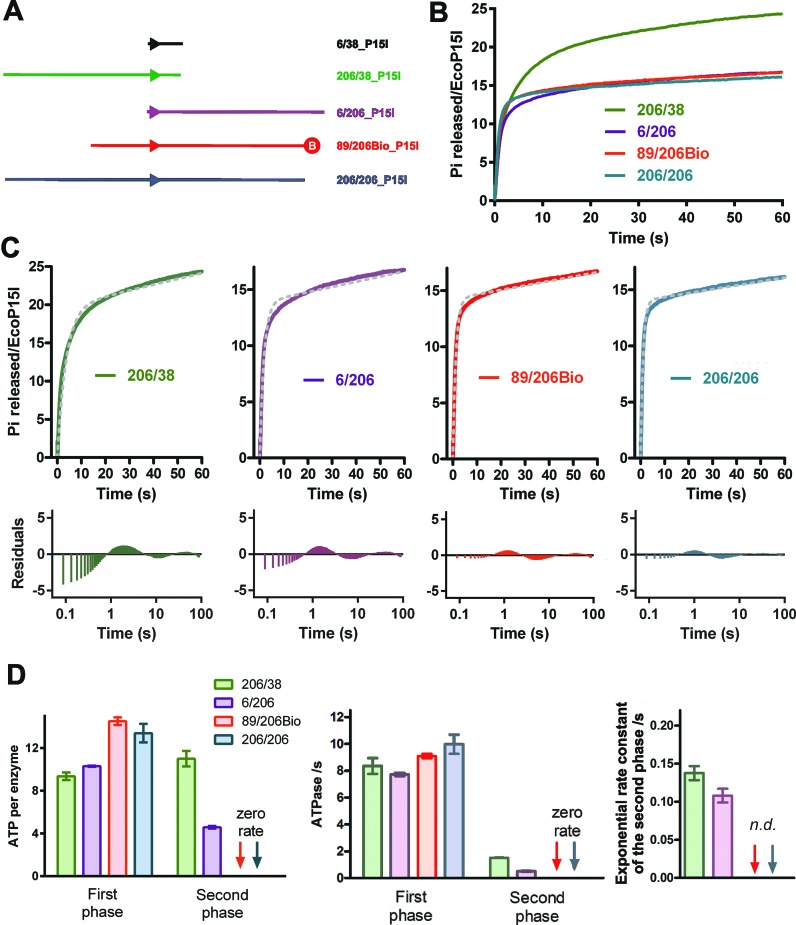
EcoP15I ATPase kinetics using longer DNA substrates. (**A**) Cartoons of the oligoduplex and longer DNA substrates. Biotin is represented as a red circle. (**B**) Preformed EcoP15I–DNA complexes (25 nM) as indicated were rapidly mixed with 4 mM ATP and 200 μM heparin, and the release of phosphate measured (Materials and Methods). Rebinding to the DNA was prevented by a heparin trap. (**C**) Fitting of the uncorrected ATPase profiles to Eq. (3) (upper graphs) and the residual differences between the experimental data and fits (lower graphs). Note that the residual plots are presented with a logarithmic time base to clearly show the deviations across three time decades. (**D**) Kinetic constants determined for the longer DNA substrates. Kinetic values for the first and second burst phases of the 206/38_P15I and 6/206_P15I profiles were determined using deconvolution as in Figure 2. Second phase kinetics could not be readily deconvoluted for 89/206Bio_P15I or 206/206_P15I (n.d. = not determined). Numbers of ATPs consumed were determined using Eq. (3). ATPase rates were determined using a linear fit (not shown). Averages and standard deviations were calculated from three repeats.

We monitored phosphate release during the ATPase cycle using the phosphate binding protein (PBP) labelled with *N*-[2-(1-maleimidyl)ethyl]-7-(diethylamino)coumarin-3-carboxamide (MDCC) as previously described (4). Fluorescence intensity measurements were performed at 25 ± 0.1°C using an SF61-DX2 stopped-flow apparatus. In all ATPase measurements, PBP-MDCC was added to both syringes at 6 μM. On each day, prior to making experimental measurements, the response of the PBP was calibrated using a titration of phosphate standard (Supplementary Figure S1A) (4). The change in the phosphate concentration during the experiments was <1 μM and was thus within the linear range. Reactions were initiated by mixing protein-DNA from one syringe with ATP and heparin from the other. For experiments in Figures 2 and 6, final reaction conditions were 200 nM DNA, 25 nM EcoP15I or EcoPI, 4 mM ATP and 200 μM heparin in Buffer R+ (50 mM Tris–HCl pH 8.0, 50 mM KCl, 10 mM MgCl_2_, 1 mM DTT, 100 μg/ml BSA). For experiments in Figure 3, final reaction conditions were: 50 nM DNA, 25 nM EcoP15I, 4 mM ATP and 2.5 μM heparin in Buffer R+.

Burst phase profiles were deconvoluted as follows and by fitting the functions to the traces using GraphPad Prism (Supplementary Figure S1B–D). Firstly, the background steady-state rate at times (*t*) > 45 s was fitted by linear regression and subtracted from the raw data. The second burst phase of the corrected profile was fitted to an offset single exponential function for *t* > 3.5 s:

(1) \begin{equation*} y = (A_2 \cdot (1 - e^{ - k_2 \cdot t} )) + A_1 \end{equation*}

where *A*_1_ is the y-axis intercept and is taken as the amplitude of the first burst phase, *A*_2_ is the exponential amplitude and is taken as the amplitude of the second burst phase, *k*_2_ is the exponential rate constant of the second burst phase. The exponential component can be subtracted from the corrected profile to give a visual representation of the burst phase. We have previously shown that *k*_2_ is very close in value to the measured single exponential rate constant for dissociation from an oligoduplex substrate (4). In other words, the second exponential burst represents the switch from an ATP hydrolysing state (at the site) to a non-hydrolysing state (sliding or dissociated from the DNA). If we assume a constant ATP hydrolysis rate prior to the start of sliding, the ATPase rate of the second burst phase can then be estimated from the number of ATPs consumed during the lifetime of the second phase, using:

(2) \begin{equation*} Rate = A_2 \cdot k_2 \end{equation*}

The ATPase rate of the first burst phase was estimated from a linear fit to the initial part of the profile (for *t* < 0.35 s).

To compare profiles without a clear second ATPase phase, the data was fitted to:

(3) \begin{equation*} y = (A_1 \cdot (1 - e^{ - k_1 \cdot t} )) + m \cdot t \end{equation*}

where *k*_1_ is the exponential rate constant of the first burst (an approximation of the lifetime of the state), *A*_1_ is amplitude of the burst phase, and *m* is the background steady-state ATPase rate. All errors we report are based on average values from separate fits to repeat data and are not standard errors of fits to averaged data.

### Tryptophan fluorescence assay

**Figure 4.**
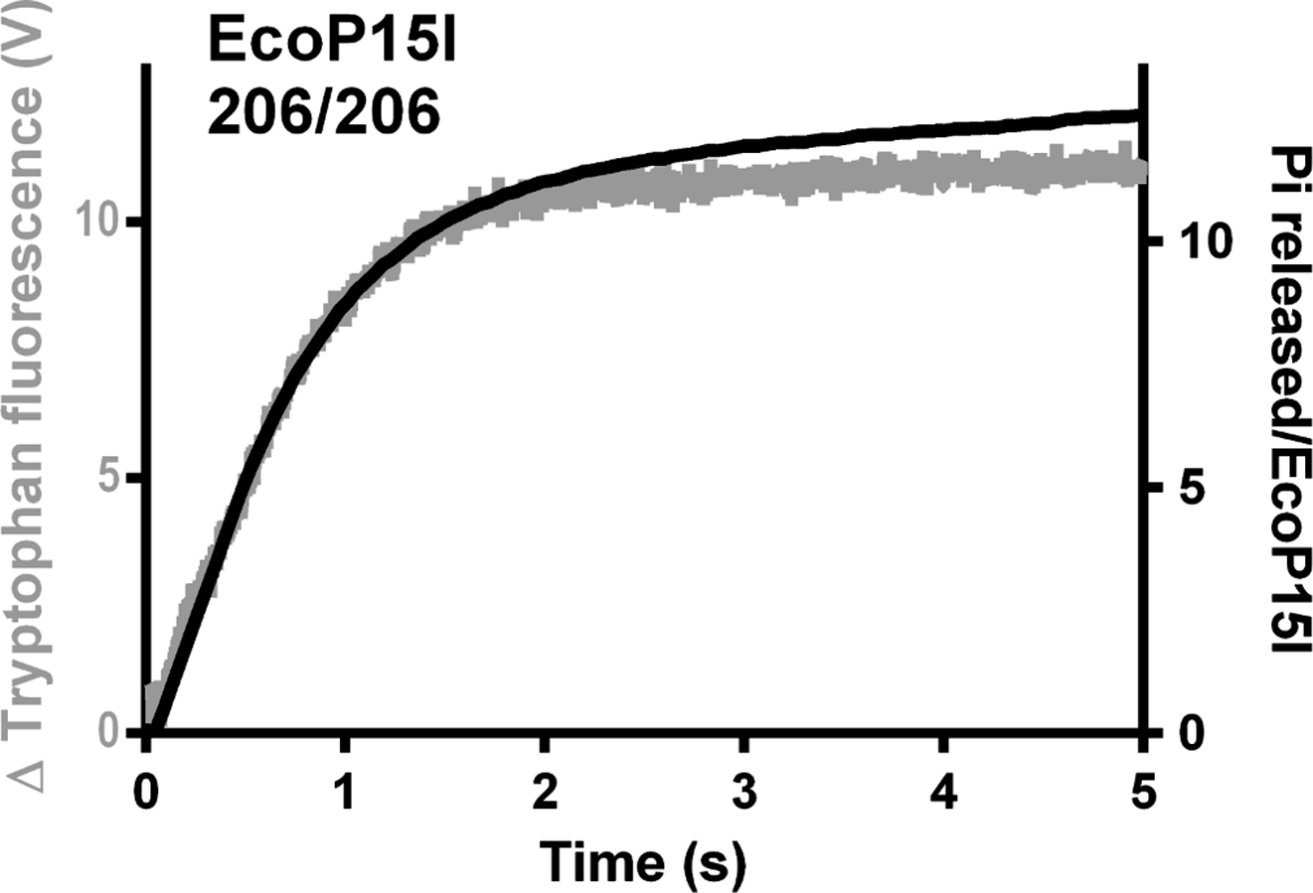
The ATPase burst kinetics match the change in tryptophan fluorescence measured using a longer DNA substrate. Comparison of the complete ATPase profile (black) from Figure 3C with the tryptophan fluorescence change observed during EcoP15I dissociation from 206/206_P15I (grey). For the tryptophan fluorescence experiment, final reaction conditions were 25 nM 206/206_P15I, 50 nM EcoP15I, 4 mM ATP and 2.5 μM heparin.

We monitored conformational changes during the ATPase cycle using tryptophan fluorescence intensity measurements as previously described (4). Experiments were performed at 25 ± 0.1°C using the SF61-DX2 stopped-flow, with *λ*_ex_ = 297 nm (4 nm bandwidth) and a 320 nm (Schott WG320) band-pass filter. Reactions were initiated by mixing protein–DNA from one syringe with ATP from the other. For experiments in Figure 2, final reaction conditions were: 1 μM DNA, 250 nM EcoPI, 4 mM ATP and 500 μM heparin in Buffer R (50 mM Tris–HCl pH 8.0, 50 mM KCl, 10 mM MgCl_2_, 1 mM DTT). For the experiment in Figure 4, final reaction conditions were: 25 nM DNA, 50 nM EcoP15I, 4 mM ATP and 2.5 μM heparin in Buffer R.

### Surface plasmon resonance assay

SPR experiments were carried out on a Biacore T200 instrument (GE Healthcare) using streptavidin coated Series S sensor SA chips (BR-1005–31), at a data collection rate of 10 Hz. Biotin-labelled DNA (89/206Bio_P15I) was immobilized onto the surface by injection of 20–50 nM DNA in Buffer R+ supplemented with 0.5 M NaCl and using Buffer R supplemented with 0.05% (v/v) Tween 20 as the running buffer, at a flow rate of 5 μl/min for 20–25 min. An in-series control channel without DNA immobilized was used for background correction in case any enzyme bound to the dextran matrix non-specifically, and also for subtracting any bulk refractive index change during nucleotide/salt injections.

**Figure 5.**
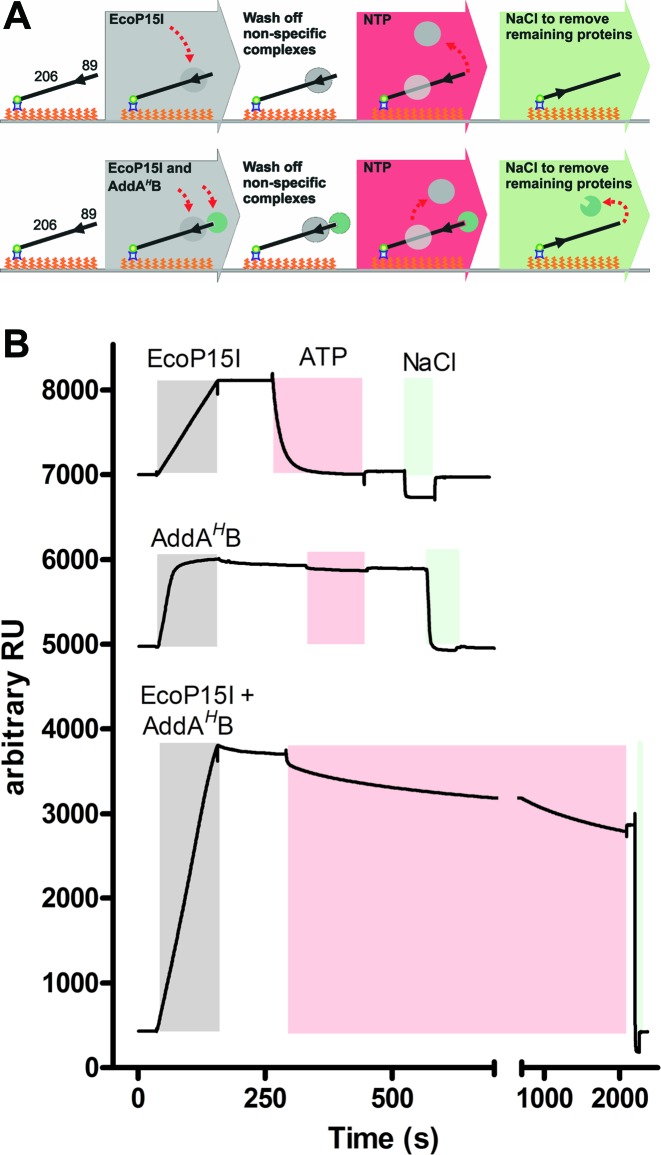
The surface plasmon resonance assay to measure DNA association and dissociation by Type III restriction-modification enzymes. (**A**) Cartoon of the steps in the assay. The DNA used was 89/206Bio_P15I. (**B**) Representative changes in response units (RU) during protein association and dissociation. Coloured blocks show the continuous injection of the enzymes (grey), ATP (4 mM, red) or NaCl (green).

Experiments were performed at 25 ± 0.1°C using Buffer R+ as the running buffer. To prevent problems with BSA precipitation, an in-line filter was used on the buffer inlet and the buffer changed after 4 h use. For the association phases, 10–25 nM EcoP15I in running buffer was injected for 120 s at 30 μl/min. In end-capping experiments, 20 nM AddA^*H*^B was co-injected with the Type III enzyme. Following protein-DNA association, non-specific complexes were allowed to dissociate in running buffer. Once a steady-state level of binding was reached, 4 mM nucleotides or nucleotide analogs (ATP, ADP or AMP-PNP) were injected at 30 μl/min in running buffer. Where indicated, 2.5 μM heparin was added to act as a trap for dissociated enzyme. For the short ATP-pulse experiments in Figure 7, the injection rate was increased to 100 μl/min. After each experimental cycle, the surface was regenerated using an injection of 1 M NaCl. Traces are averages of three repeats, except for the data in Figures 5B, 7B and E where single measurements are shown. Data was analysed by fitting the traces to single exponential decay functions using GraphPad Prism (4).

## RESULTS AND DISCUSSION

### EcoPI has a two phase ATPase cycle on an oligoduplex similar to that seen with EcoP15I, but with different apparent efficiencies in ATP coupling

To explore whether the EcoP15I-DNA interaction was unusual in generating a dual phase ATPase profile, we first investigated the activity of EcoPI on an oligoduplex substrate with the same up- and downstream DNA sequence (Figure 2A). EcoPI is homologous to EcoP15I; the Res subunits (containing the helicase-like and nuclease domains) share ∼90% identity whilst the Mod subunits (containing the methyltransferase and DNA target recognition domains) share >60% identity (with most differences being between the Target Recognition Domains - TRDs) (12). Despite the similarities at the sequence level we and others have noted some differences in enzyme properties (9,13–16). We analysed EcoPI using millisecond time resolution stopped flow assays previously applied to EcoP15I (4): ATPase activity was measured using a fluorescent phosphate binding protein; changes in protein conformation were monitored using tryptophan fluorescence (Materials and Methods). Binding and dissociation of EcoP15I from its oligoduplex was previously measured using a fluorescence anisotropy assay (4). Unfortunately, we were unable to obtain a measurable signal change with this assay using EcoPI.

The ATPase reactions were initiated by rapidly mixing a pre-incubated, saturated EcoPI–oligoduplex complex with ATP and heparin (the latter as a trap to capture dissociated enzymes and thus create single turnover kinetic conditions with respect to DNA binding). Incomplete trapping and DNA rebinding results in a linear steady-state rate which needed to be corrected before further analysis (Supplementary Figure S1B). The kinetics can be deconvoluted into a rapid burst phase and a slower exponential burst phase (Materials and Methods). The corrected phosphate release profile shows clear evidence for two kinetics phases analogous to those seen previously with EcoP15I (Figure 2B). The amplitudes of the first and second bursts—how much ATP is consumed during those states—could be estimated from the ordinate intercept and amplitude of the exponential phase, respectively (Eq. (1), Supplementary Figure S1C). The rate constant gave a measure of the lifetime of the state (before it switches to DNA sliding). Therefore, a linear ATPase rate for the second phase could be estimated based on the lifetime and the number of ATPs consumed during the lifetime (Eq. (2)). Note that the observed rate is actually decaying as the enzyme dissociates from its recognition site. The ATPase rate of the first phase was estimated by a linear fit (Supplementary Figure S1D).

The rate and amplitude of the first EcoPI burst phase were very similar to values seen with EcoP15I (Figure 2C). We also found that the kinetics of the EcoPI conformation change as measured by tryptophan fluorescence (Figure 2D) showed a close correspondence with the kinetics of the first burst (Figure 2E), as seen for EcoP15I (4). However, the kinetics of the second phases were quite different, with EcoPI consuming a third as much ATP at a five-fold slower rate.

We showed previously for EcoP15I that the rate of the exponential, second burst phase corresponded to the rate-limiting dissociation from the DNA (which given the rapid 1D sliding and short DNA length corresponded to the time to escape from the site) (4). Although we could not measure the dissociation kinetics of EcoPI directly, we could use the exponential, second burst phase of the EcoPI ATPase profile to infer that the lifetime at the site is ∼11.5 s, which is almost twice that of EcoP15I (Figure 2C). This is surprising as previous results suggest that EcoPI is more weakly bound than EcoP15I (9). The slower dissociation could be due to the slower observed ATPase rate. Nonetheless, the difference between EcoPI and EcoP15I in the number of ATPs consumed in the second burst phase would imply that this stage in the process does not require a fixed number of coupled ATP hydrolysis steps.

### The kinetics of ATP hydrolysis by EcoP15I is influenced by the proximity of DNA ends to the site

In the previous EcoP15I experiments, we chose to use a short oligoduplex substrate (Figure 1A) so that we could readily compare the ATPase and tryptophan fluorescence data with an anisotropy-based DNA dissociation assay (4). To examine if the DNA length was affecting the observed enzyme activity, we measured the EcoP15I ATPase kinetics using longer linear DNA (Figure 3A), where the site was either located centrally (206/206_P15I), or at distal positions where either the upstream DNA length (6/206_P15I) or downstream DNA length (206/38_P15I) matched those in the oligoduplex (6/38_EcoP15I, Figure 1A). Additionally, we tested a DNA substrate used subsequently in the SPR assay (89/206Bio_P15I - see below). The ends of the DNA were left free of protein roadblocks (i.e. ‘uncapped’), so that sliding enzymes could dissociate and be trapped by heparin, so giving single turnover burst kinetics.

The phosphate release profiles clearly show that proximal DNA ends affect the ATPase kinetics (Figure 3B-D). The profile for 206/38_P15I was clearly biphasic and similar to that observed with 6/38_P15I (4), albeit with apparent differences in rates/amplitudes (Figures 2C and 3D). In contrast, both 206/206_P15I and 89/206Bio_P15I showed ATPase reaction profiles that differed in appearance to the clear biphasic profiles of 6/38_P15I and 206/38_P15I. The 6/206_P15I DNA showed an intermediate profile, where a second phase was noticeably smaller than with 206/38_P15I but still more visibly present than the DNA with more centrally-located sites. It therefore appeared that the location of DNA ends both up- and downstream of the EcoP15I site in the oligoduplex could influence the shape of the observed ATPase profiles.

For 6/206_P15I and 206/38_P15I, where the reaction profile could be clearly split into two phases, we were able to apply the same deconvolution process as above (Materials and Methods). However, for 206/206_P15I and 89/206Bio_P15I, we were unable to identify and fit satisfactorily a second burst phase. To compare the different profiles, we attempted to fit the uncorrected data directly to a single exponential plus steady state linear (to account for a low steady state which likely reflects DNA rebinding due to the leakage from the heparin trap—Eq. (3), Materials and Methods) (Figure 3C). For the 6/206_P15I and 206/38_P15I profiles, plots of the residual differences between the fitted and experimental data showed clear systematic deviations, particularly during the first burst phase. The deviations were largest for 6/206_P15I which is more plainly biphasic. For 206/206_P15I and 89/206Bio_P15I we still observed systematic deviations but these were smaller than the other substrates. Since we could not use the deconvolution process for these DNA, we used the amplitude of the exponential fit to estimate ATP consumption. The initial ATPase rate was estimated from a linear fit, as above (Materials and Methods).

The amplitudes and rates determined for the longer DNA are compared in Figure 3D. The observed rates of the first burst phases were similar for all the DNA, but with an elevated burst amplitude where the DNA ends were more distantly located from the site. In contrast, the ATPase rates and amplitudes of the second burst phase varied noticeably with the position of the site. For 6/206_P15I and 206/38_P15I, the rates of the second burst phase exponential were similar to that measured for the oligoduplex (Figures 2C and 3D). We could interpret this as showing that the rate of change from an ATP hydrolysing state at the site to a non-hydrolysing sliding state is similar on all these DNA. However, the number of ATPs consumed during this process varies from ∼13 to ∼5, dependent upon proximity of DNA ends. For 206/206_P15I and 89/206Bio_P15I we considered the rates and amplitudes of the second phases as nominally zero in Figure 3D, although we note that we cannot completely exclude the hydrolysis of one or two ATPs after the first phase due to detection limits in our assay. Being distant from a DNA end largely eliminates the consumption of ATP in the second phase. Despite not having a measurable second ATPase burst, we demonstrate below that dissociation is as rapid on 89/206Bio_P15I as on the oligoduplex. For the same reason we do not consider the linear steady-state phase as representing a slow second ATP phase associated with slow DNA dissociation.

We also measured the change in EcoP15I tryptophan fluorescence using 206/206 as a substrate (Figure 4). The kinetics correlate closely with the first ATPase burst, as seen previously using the oligoduplex substrate (4). These data suggest that the single phase of ATPase activity seen on 206/206_P15I and 89/206Bio_P15I is sufficient to generate and maintain the conformational change. It appears that ATP hydrolysis following the conformation change is only observed when a downstream DNA end is proximal to the recognition site.

### A surface plasmon resonance assay to measure dissociation of EcoP15I from uncapped and capped DNA

To correlate the ATPase and conformation change kinetics in Figures 3 and 4 with initiation of DNA sliding, we developed an assay to follow DNA association and dissociation compatible with longer DNA substrates (Materials and Methods). We used an SPR-based assay which allows dissociation from DNA bound to the chip surface (via a biotin–streptavidin linkage) to be followed in real time and which also allows rapid exchange of buffer components (Figure 5A) (17,18). Reactions were corrected for background binding using an in-series reference surface without DNA (Materials and Methods). Compared to the oligoduplex anisotropy assay (4), the SPR assay has the advantage that we can monitor linear DNA of longer lengths and that we can readily cap the free end of the DNA to observe the effect of only allowing endo-dissociation. A DNA substrate with a single EcoP15I site (89/206Bio_P15I) was made by PCR using one primer labelled with biotin (Figure 3A) (Materials and Methods). Binding of the DNA to the streptavidin/dextran surface of the chip caps one end of the DNA, preventing dissociation during sliding via this end; the opposite end was either uncapped or capped with a protein (see below). Following the initiation of sliding by ATP hydrolysis, exo-dissociation from uncapped 89/206Bio_P15I would take <3 ms (4,10). Therefore, we can still use the rate of DNA dissociation via an end as a proxy for the rate of site release into the sliding. In other words, any observed differences between the dissociation rates from 6/38_P15I compared to uncapped 89/206Bio_P15I would reflect different events at the site, rather than differences in sliding lifetime.

In a typical experiment (Figure 5A, *upper panel*), enzyme was injected over the chip to bind the DNA. Buffer flow was continuous, allowing dissociation and equilibration of binding to a steady-state. Following a wash step, dissociation is then initiated by injection of nucleotides such as ATP (at 4 mM). Finally, the DNA-chip is regenerated using NaCl and further experiments can be made. To measure dissociation from capped DNA (Figure 5A, lower panel), we also included a helicase mutant of the end-processing enzyme AddAB (AddA*^H^*B), in which the Walker A motif of AddA has been mutated (K36A) to prevent ATP hydrolysis (Materials and Methods) (11,19). AddA*^H^*B binds tightly and specifically to DNA ends and is unaffected by ATP (11,19). Dissociation of EcoP15I from this DNA must be via endo-dissociation.

Representative raw SPR profiles plotted as response units versus time are shown in Figure 5B (attempts to repeat similar assays using EcoPI were unsuccessful due to increased background noise and low response signal):

- *EcoP15I association and dissociation from uncapped DNA*. Injection of EcoP15I caused an increase in response units corresponding to specific DNA binding at the recognition site. The protein–DNA complex formed was relatively stable over the time course of the reaction. Subsequent injection of 4 mM ATP for 180 s caused rapid and near-complete dissociation from the DNA within ∼60 s. Addition of the nucleotide caused a reproducible refractive index shift in the response units, suggesting a difference in background response for the reference and reaction surfaces. Enzymes that remained associated with the DNA and/or chip were removed by the salt wash, allowing the reaction to be repeated (again, note the observed drop in response units during salt injection due to a refractive index correction anomaly).
- *Stable binding of AddA^H^B to DNA in the presence of ATP*. Injection of AddA*^H^*B caused an increase in response units corresponding to DNA binding. A small burst of dissociation leading to a steady state was observed following the switch to buffer alone. Although injection of ATP resulted in a refractive index shift, following the switch back to buffer, the level of AddA*^H^*B binding was near constant. AddA*^H^*B association with the DNA could be subsequently reversed by the salt wash.
- *EcoP15I association and dissociation from capped DNA*. Simultaneous injection of EcoP15I and AddA*^H^*B caused an elevated increase in response units corresponding to DNA binding by both enzymes (and equivalent to the sum of response unit changes for consecutive enzyme injections). Following switch to running buffer, a small re-equilibration was observed due to AddA*^H^*B dissociation. Subsequent injection of ATP caused significantly slower protein dissociation from the DNA, consistent with slower endo-dissociation of EcoP15I (4,20). The actual percentage of DNA bound could be corrected by subtracting the response units corresponding to AddA*^H^*B. Any enzymes remaining bound at the end of the reaction could be dissociated using the salt wash.

**Figure 6.**
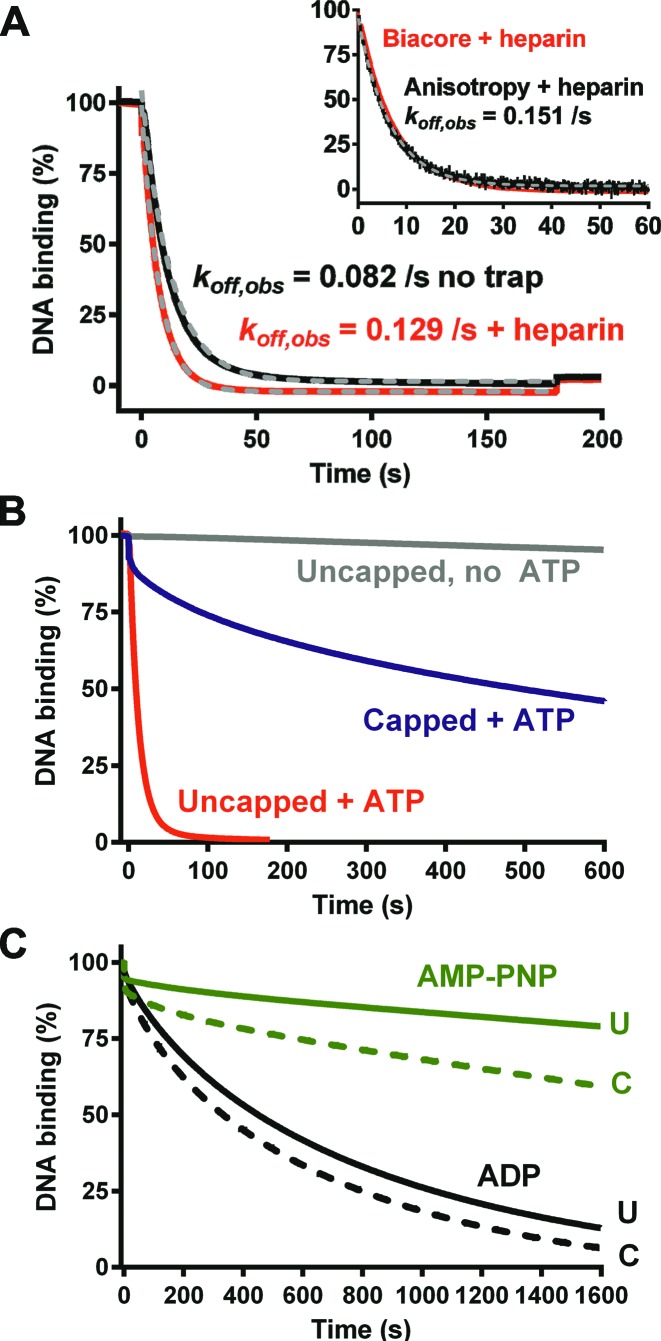
Dissociation of EcoP15I from DNA measured using the surface plasmon resonance assay. (**A**) (Main graph) EcoP15I-DNA dissociation driven by injection of 180 s of ATP in the presence or absence of heparin. Grey dashed lines and associated rate constants are single exponential fits to the data. (Inset) Comparison of the dissociation measured by the SPR assay in the presence of heparin to dissociation measured from hexachlorofluorescein-labelled 6/38_P15I using the anisotropy assay (data from (4)). Grey dashed line and associated rate constant is a single exponential fit to the anisotropy data. (**B**) Comparison of EcoP15I-DNA dissociation from uncapped or capped DNA under different conditions. Injection times were 180 s (ATP on uncapped DNA) or 600 s. (**C**) Comparison of EcoP15I–DNA dissociation from uncapped DNA (U, solid line) or capped DNA (C, dashed line) upon injection of either AMP–PNP or ADP for 1600 s.

The dissociation profile from uncapped DNA measured using the SPR assay could be fitted to a single exponential decay, with a rate constant of ∼0.08/s (Figure 6A). This value appeared slower than that measured using the anisotropy assay (∼0.15/s) (4). As one of the binding partners has to be immobilized, SPR measurements can be affected by the mass transfer effect (21–23). To bind the immobilized ligand, the analyte firstly has to transfer from bulk solution to the chip surface via diffusion and convection. Where this transfer is slower than the analyte-ligand association rate, and the dissociation rate is slow, the solution near the interaction surface can become depleted of free analyte. This can limit both the observed on- and off rates. To test if our measurements were affected by mass transfer, we measured EcoP15I dissociation at flow rates between 5–100 μl/min (data not shown). Mass transfer is influenced by the flow rate whereas the intrinsic reaction rate is not. For all the measurements in this work, we applied a flow rate of 30 μl/min where the off rate became saturated. In addition, we reasoned that the observed rate may be slower because following ATP addition and DNA dissociation, enzymes were re-associating with the immobilized DNA. To counter this, we added heparin in the running buffer to trap dissociated enzyme. Accordingly, the dissociation observed had a single exponential rate very similar to that from the anisotropy assay (Figure 6A). Unfortunately, we found that addition of the heparin trap caused significant problems in run-to-run reproducibility. Subsequent reactions were therefore undertaken without heparin unless stated and thus must be compared to the slower apparent dissociation rate.

The similarity in the dissociation kinetics between long and short DNA (Figure 6A) rules out that the observed differences between the kinetic parameters of the second ATPase burst in Figures 2C and 3D are correlated with changes in the rate of dissociation. i.e. the kinetics of site release are the same for both DNA. Instead it would appear that the second phase of ATP hydrolysis is completely decoupled from the dissociation process, and may just be an artefact of the proximity of DNA ends in the oligoduplex (see Discussion).

We measured the dissociation from uncapped and capped DNA with a range of nucleotides (Figure 6B and C) as before (4). With ATP, dissociation from the capped DNA showed a small rapid burst, followed by a slow dissociation with a half-life in excess of 600 s. Single molecule and ensemble assays have suggested an endo-dissociation lifetime for EcoP15I and EcoPI of ∼200 s (4,20). The longer lifetime observed here most likely reflects mass-transport-dependent rebinding events in the absence of heparin. The small rapid burst may be due to a subset of enzymes that dissociate directly from the site in an ATP-dependent manner. ATP-dependent direct dissociation without extensive DNA sliding was also observed in the single molecule assay (4).

Injection of either ADP or the non-hydrolysable ATP analogue AMP–PNP produced dissociation kinetics similar to those observed previously with the anisotropy assay and using oligoduplex 6/38_P15I (Figure 6C) (4). The kinetics on the capped and uncapped DNAs were similar in each case, suggesting that endo-dissociation events predominate with these nucleotides. For AMP-PNP this is most likely because sliding does not occur and dissociation occurs directly from the site. In contrast, ADP can support long-lived DNA sliding (4). Although ADP-sliding is slower than ATP-sliding (∼0.5 × 10^6^ random single nucleotide steps per second), EcoP15I should still have reached the end of uncapped 89/206Bio_P15I within tens of milliseconds. The slow dissociation observed may be because the ADP sliding state binds tightly to DNA ends, favouring exit by endo-dissociation with a rate similar to capped DNA.

### One second of ATP hydrolysis is sufficient for DNA dissociation over at least 20 seconds

Since the SPR assay was conducted under continuous flow conditions, it allowed us to transiently introduce a reagent and then wash it away almost instantaneously with reaction buffer. The initial ATPase phase and conformational switch is completed in ∼1 s (Figures 2E, 3B and 4) (4). Within the limits of the Biacore T200 instrument, ATP can also be injected down to an incubation time of ∼1 s (Materials and Methods). Therefore, we could transiently introduce ATP to allow the majority of the first phase to be completed, instantaneously switch to running buffer without ATP to prevent any further ATP turnover, and observe the effect on dissociation. This modified assay also tested whether sliding requires ATP hydrolysis, as rapid dissociation from the surface-bound uncapped DNA required that the enzyme travelled by 1D diffusion to the single free DNA end (using millions of randomized single base pair steps).

**Figure 7.**
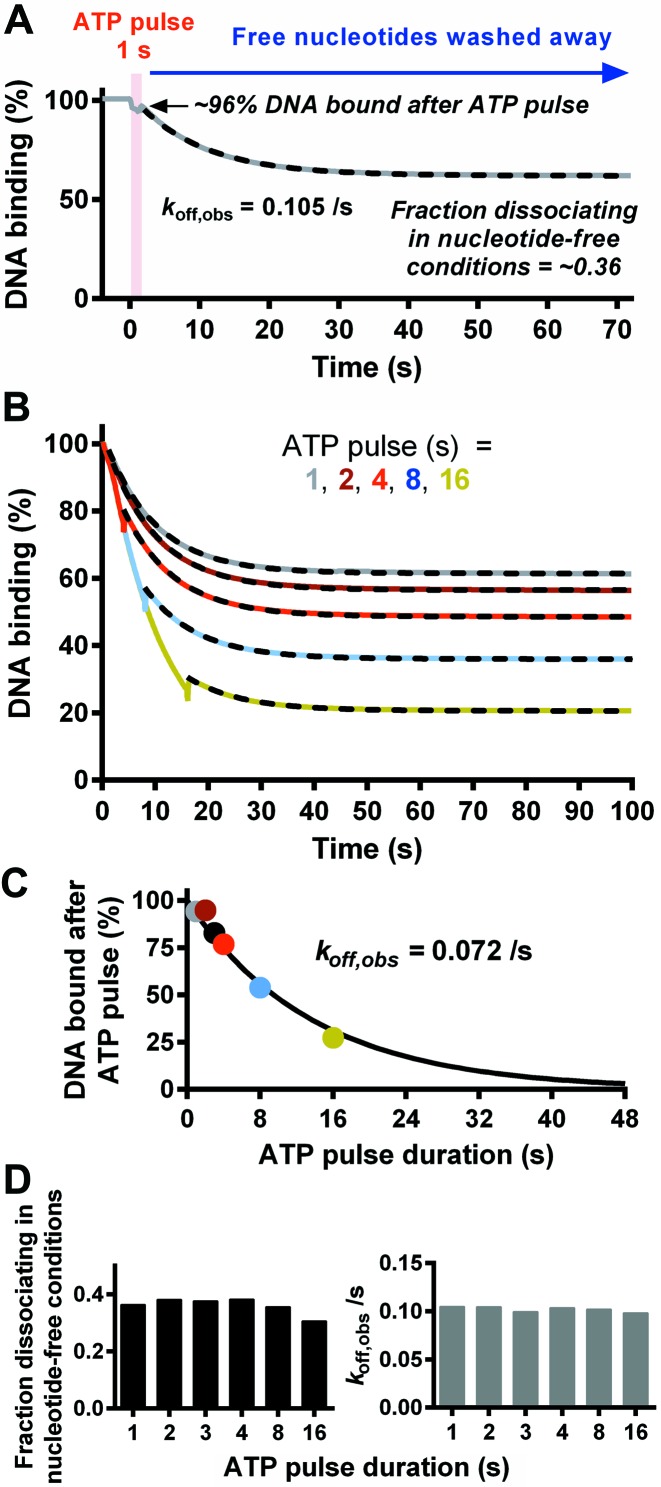
The effect of transiently introducing ATP on the dissociation of EcoP15I from DNA. (**A**) EcoP15I-DNA dissociation measured in the SPR assay was initiated by injection of ATP for 1 s (red block) followed by Buffer R+ without nucleotide. The dashed line shows a fit to a single exponential function with incomplete dissociation. (**B**) Comparison of dissociation with variations in the times of ATP injection. Dashed lines are fits of the dissociation rate to a single exponential function following return to Buffer R+. (**C**) Percentage of DNA bound following the ATP pulse, as a function of ATP pulse duration. Colours are as in panel B. The data can be fitted with a single exponential function due to the kinetics of the underlying dissociation process. (**D**) Kinetic constants determined from single exponential fits to data in panel B. (Left graph) The fraction of enzymes remaining bound after the ATP pulse that subsequently dissociated during the ATP-free period. (Right graph) The rate constant for dissociation following the return to ATP free conditions (Buffer R+).

The dissociation from uncapped DNA induced by a 1 s pulse of ATP followed by nucleotide-free buffer is shown in Figure 7A. Dissociation during the 1s pulse is masked by the refractive index anomaly (see above), but can be measured from the percentage of binding following the switch to reaction buffer. Although <4% of enzymes dissociated during the ATP pulse, a further third of the enzymes released from their DNA during the nucleotide-free phase. The dissociation in the absence of ATP followed a single exponential (dashed fitted line in Figure 7A), with a rate constant similar to that in the presence of ATP (Figure 6A). Therefore dissociation can continue at the same rate despite the absence of ATP.

The reaction was repeated with varying ATP pulse times (Figure 7B). Although the dissociation profiles during the 4, 8 or 16 s ATP pulses could be directly fitted to an exponential, and clearly overlay one another in Figure 7B, dissociation during the shorter pulses was too low to fit accurately (data not shown). Instead we plotted the percentage of DNA bound immediately after each ATP pulse against the pulse duration time (Figure 7C). The combined data follows a single exponential decay with a kinetic constant consistent with the expected rate of dissociation. Therefore changing the pulse time has not affected the observed dissociation kinetics in the presence of ATP.

The dissociation profiles in nucleotide-free conditions following each ATP pulse were individually fitted to a single exponential decay (dotted lines in Figure 7A). The amplitudes and rate constants of the fits are presented in Figure 7D. Regardless of the ATP exposure time, EcoP15I continued to dissociate from the DNA at much the same rate once ATP was removed. As noted above for the 1 s pulse, the rate of dissociation in nucleotide-free conditions was very similar to that observed when ATP was available throughout the dissociation process. The small relative decrease in the fraction of enzymes that dissociated following the 16 s pulse suggested that enzymes that remain bound to the DNA for longer times in the presence of ATP may have a greater chance of entering an irreversibly inhibited state.

**Figure 8.**
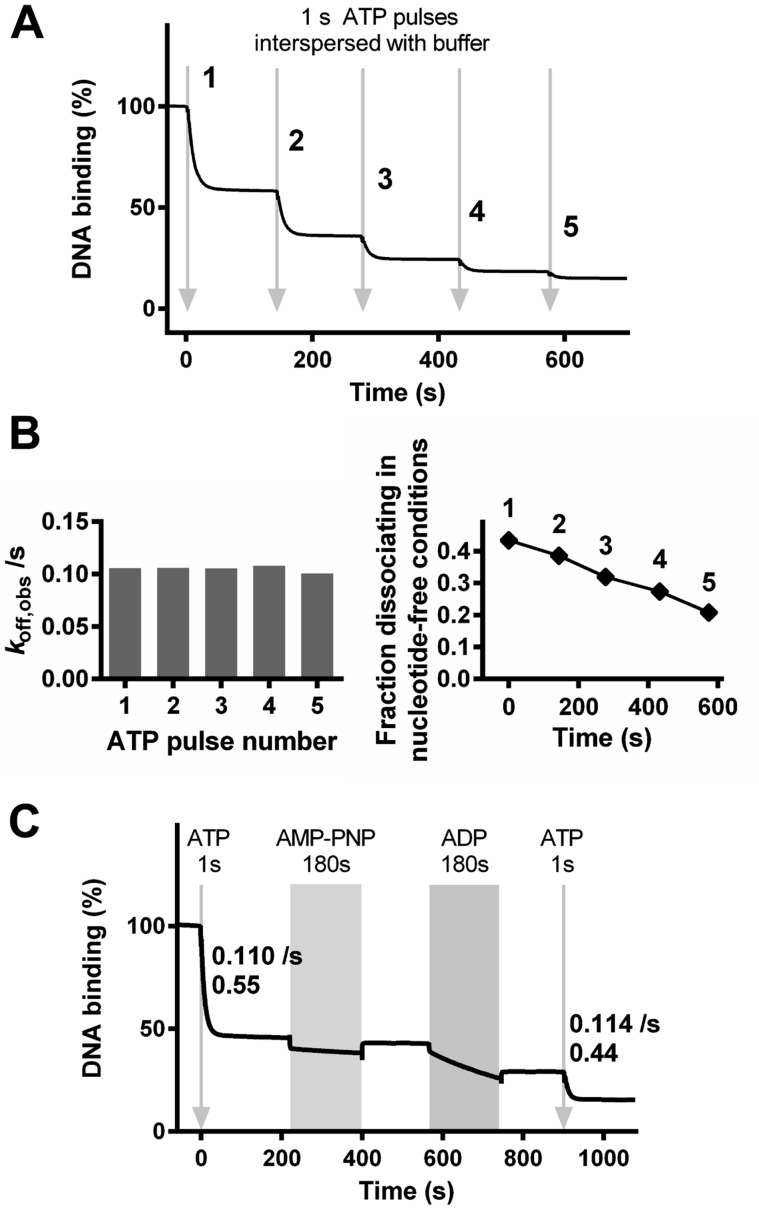
The effect of consecutive nucleotide additions on DNA association and dissociation by EcoP15I. (**A**) EcoP15I-DNA dissociation measured in the SPR assay was initiated by consecutive injections of ATP for 1 s (arrows) followed in each case by Buffer R+ without nucleotide. (**B**) Kinetic constants are shown from fits of the EcoP15I–DNA dissociation profiles following each ATP pulse in panel A to an offset single exponential function. The change in the amplitude as a function of total reaction time appears approximately linear. (**C**) EcoP15I–DNA dissociation measured in the SPR assay was initiated by consecutive injections of: ATP for 1 s (arrow) followed by Buffer R+; AMP–PNP for 180 s (grey block) followed by Buffer R+; ADP for 180 s (grey block) followed by Buffer R+; and, ATP for 1 s (arrow) followed by Buffer R+. Kinetic rate constants and fractional amplitudes are shown for the single exponential dissociation produced by each ATP pulse.

In the above experiments, dissociation did not go to completion. One reason could have been recognition site rebinding during the sliding process. Rebinding was first observed in single molecule experiments (4). The events were reversible, but ATP was present throughout these reactions. If ATP binding and/or hydrolysis were required to escape the rebound state and return to sliding, then the lack of available ATP following the pulses in Figure 7 may have prevented a return to the sliding state. An alternative reason for incomplete dissociation is that following release from the original DNA, mass-transport caused binding to new DNA. This may have been inefficient as we have evidence that EcoP15I enzymes released from a DNA end are in a structural state that cannot associate with DNA and that this state has a lifetime of tens of seconds (4). To explore the incomplete dissociation further, we observed the effects of repeated pulses of one second of ATP interspersed with running buffer (Figure 8A).

Consecutive dissociation bursts could be initiated with similar single exponential rate constants each time (Figure 8B, left panel). This suggests that at least a subset of the enzymes have rebound to a site (either on the original DNA or on a new DNA) and dissociation can be re-initiated by the introduction of ATP. The fraction of enzymes dissociating dropped with each successive injection however (Figure 8B, right panel), suggesting that some of the enzymes accumulated in an irreversibly inhibited state, either on the DNA or on the surface. This inhibition does not appear to follow a simple single exponential relationship with time. To test if the binding of other nucleotides could re-start sliding by the rebound enzymes, we introduced a 1 s pulse of ATP, allowed equilibration to a steady state, and then followed with 180 s pulses of AMP–PNP and ADP (Figure 8C). Each nucleotide produced dissociation kinetics similar to that seen for EcoP15I bound to DNA without having seen ATP (Figure 6C). For both nucleotides, the dissociation stopped as soon as the injection finished. A second 1 s pulse of ATP produced the expected dissociation kinetics (Figure 8C).

## CONCLUSIONS

We previously measured a dual ATPase cycle during the initiation of sliding by EcoP15I on a short oligoduplex (4). We show here that this is not a unique feature of EcoP15I since EcoPI also had a dual ATPase cycle on an oligoduplex albeit with different kinetics for the second burst phase. However, we also demonstrate that the amplitude and rate of the second ATPase phase is dependent upon the nature of the DNA substrate and, most likely, dispensable for the initial initiation of sliding. Although we cannot completely rule out that a few ATPs are hydrolysed in a second phase, we disfavour the models in Figure 1. Instead we suggest that the initiation of DNA sliding by Type III RM enzymes requires a single phase during which multiple ATPs are hydrolysed leading to a conformation switch; further ATP hydrolysis is not necessary for the switch, for dissociation from the site or for the sliding process.

The second phase of ATP hydrolysis seen with oligoduplexes, both here and previously (4), appears to be due to the presence of DNA ends in proximity to the complex. Recently Gupta *et al*. published the co-crystal structure of a Res_1_Mod_2_ heterotrimer of EcoP15I bound to its specific recognition site on a DNA with 3 bp upstream and 11 bp downstream (24). It is possible that extending the upstream DNA by 3 bp will allow an interaction with the ModB subunit via its TRD, which is swung away from the DNA in the structure (the TRD of ModA making all the specific recognition contacts at the site). However, the upstream end had a rather modest effect in producing an extended second phase compared to proximity of the downstream end. Surprisingly, on the 6/38 oligoduplexes and the longer 206/38 DNA, the downstream end is 11 bp from the nearest cut site, where the nuclease domain must bind, and is ∼27 bp away from where the helicase-like domains interact. Since the nuclease domain is not fully resolved in the structure (24), we cannot confidently speculate on where or how the interaction may occur. It is possible, for example, that the DNA wraps around the nuclease domain so that the downstream end interacts with the complex despite being almost four DNA turns from the site. We suggest that while future studies of Type III enzymes could empirically determine the effect of local DNA ends, a more simple solution will be to use DNA substrates where the Type III sites are placed sufficiently far away from the ends that they do not influence the kinetics.

Consistent with the necessity for only a single burst of ATP hydrolysis and with a sliding state that does not consume ATP, DNA dissociation in the SPR assay continued for up to sixty seconds after a one second pulse of ATP. However, compared to sliding in the continued presence of ATP, dissociation was incomplete. The un-dissociated proteins appear to return to their original recognition site-bound states, as inferred from the effects of subsequent additions of either ATP or other nucleotides. Fast dissociation could be reinitiated by further ATP treatment but not by a non-hydrolysable ATP analogue, suggesting that another burst of ATP hydrolysis might be required. This contrasts with previous data where the ATP hydrolysis kinetic profiles on capped and uncapped DNA were similar, suggesting that re-binding to the site observed directly by single molecule imaging does not induce significant hydrolysis (4). Continued ATP *binding* after the conformation switch may play an important role in maintaining the sliding state, particularly when the enzyme re-visits a site. When ATP is removed following the switch, the conformation may be able to relax to a non-sliding state at the site, allowing essentially irreversible rebinding. The diffusion coefficient may also be altered.

The EcoP15I-DNA structure was solved from crystals grown in the presence of AMP (24). Therefore, the protein is likely to be in the pre-switch, non-sliding state. It was speculated that ATP hydrolysis causes a 40° rotation of the TRD of ModA so that it can release the recognition site and directly contact the Pin domain of the C-terminal RecA fold of the helicase-like domain (RecA2). This would require rearrangement of the helicase-like domain which may prevent further ATP hydrolysis. However, it is possible that ATP may still be able to bind in the cleft in a non-hydrolytic manner, to help maintain the TRD–pin interaction. We have also observed here, and previously (4), variable effects of other nucleotides on dissociation and formation of sliding conformations. These in turn may reflect alternative arrangements of the helicase-like domains. It would be informative to solve the structures of alternative co-crystals: nucleotide-free, with ADP, with non-hydrolysable nucleotide analogues, and post-hydrolysis of ATP.

Although the data here simplifies the ATPase scheme of the Type III RM enzymes to a single catalytic burst of ATP hydrolysis, it still remains unclear why multiple ATPs are consumed to produce the conformation change in the complex. Although other groups have presented data to support linear stepwise translocation over longer distances (25–28), we have yet to find evidence for such movement during the initiation process or during long-range communication itself (3,5,9,20). Instead we have speculated that the reason why multiple ATPs are consumed is due to mechanochemical uncoupling (6), where the helicase releases ADP/phosphate before the conformational switch can complete and another round of ATP binding/hydrolysis must take place to maintain the stressed state. The crystal structure (24) now provides a framework to further investigate the questions of why multiple ATPase cycles are required, and of how the Res and Mod subunits rearrange to produce a very efficient DNA sliding machine.

## Supplementary Material

SUPPLEMENTARY DATA
