# Supplementary material for: Re-evaluating the kinetics of ATP hydrolysis during initiation of DNA sliding by Type III restriction enzymes
Source: Nucleic Acids Res. 2015 Nov 3;43(22):10870–81. doi: 10.1093/nar/gkv1154 (PMC4678819; doi:10.1093/nar/gkv1154)
Supplement: SUPPLEMENTARY DATA [file supp_43_22_10870__index.html]

Re-evaluating the kinetics of ATP hydrolysis during initiation of DNA sliding by Type III restriction enzymes — SUPPLEMENTARY DATA 

# Re-evaluating the kinetics of ATP hydrolysis during initiation of DNA sliding by Type III restriction enzymes

## SUPPLEMENTARY DATA

- SUPPLEMENTARY DATA
