# Supplementary material for: Re-evaluating the kinetics of ATP hydrolysis during initiation of DNA sliding by Type III restriction enzymes
Source: Nucleic Acids Res. 2015 Nov 3;43(22):10870–81. doi: 10.1093/nar/gkv1154 (PMC4678819; doi:10.1093/nar/gkv1154)
Supplement: SUPPLEMENTARY DATA [file supp_gkv1154_nar-02633-h-2015-File010.pdf]

Supplementary data to:

Re-evaluating the kinetics of ATP hydrolysis during initiation of DNA sliding by Type III restriction enzymes

Júlia Tóth, Jack Bollins and Mark D. Szczelkun

DNA-Protein Interactions Unit, School of Biochemistry, University of Bristol, Bristol, BS8 1TD, UK

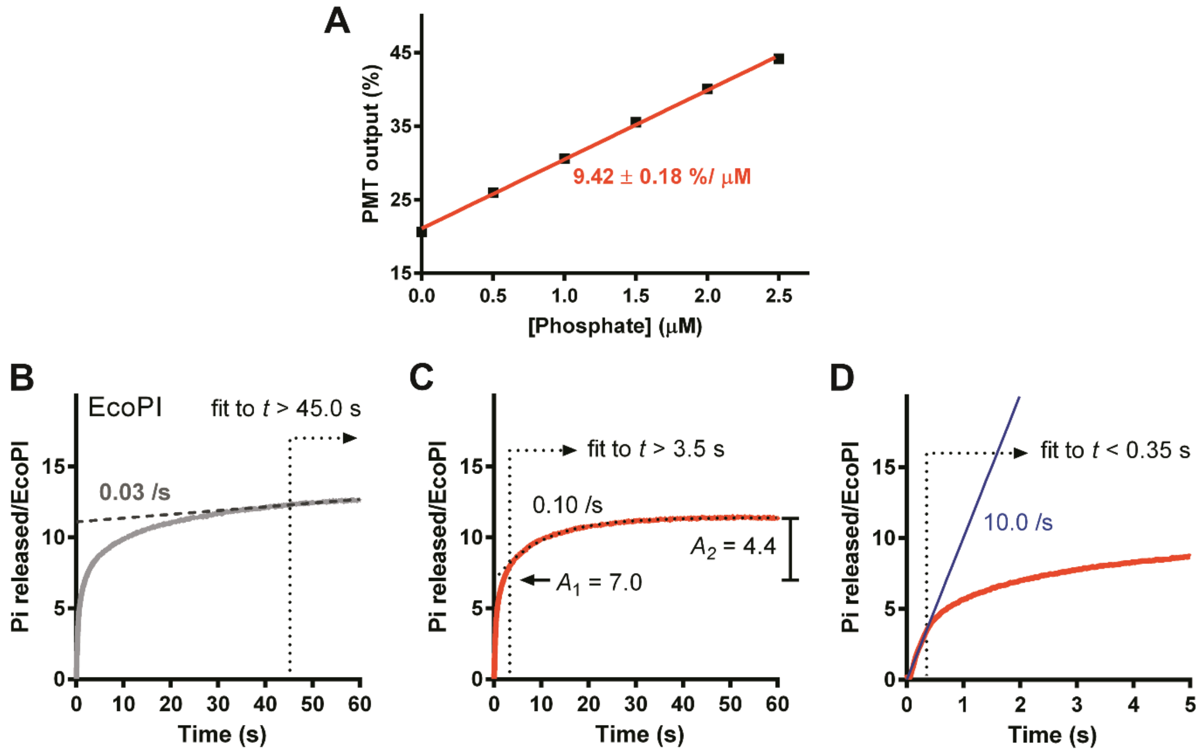

Supplementary Figure S1. The phosphate release assay and deconvolution of the ATPase kinetics. (**A**) Representative calibration of the phosphate binding protein (PBP). 6 μM PBP (final, post-mix concentration) was mixed with the concentrations of phosphate standard as shown and the output of the photomultiplier recorded. Linear regression was used to estimate the fluorescence response. (**B – D**) Example of the deconvolution of the EcoPI ATPase kinetics. The background steady-state rate of ATP hydrolysis that results from binding of EcoPI to heparin was estimated using linear regression to the raw data (grey line in B) for t > 45.0 s. The gradient was used to correct the raw data. Note that the rate is shown as phosphate released/EcoPI whereas the background rate actually comes from heparin binding. The corrected data (red line in C) was fitted for t > 3.5 s to equation 1 (dotted line in C), to estimate the ATP amplitudes for the first (A_1) and second (A_2) ATPase bursts. The exponential rate constant can be used as a measure of the lifetime of the second burst (see main text). The corrected data (red line in D) was fitted for t < 0.35 s using linear regression to estimate the initial ATPase rate of the first burst. Note that in panel D the X-axis is zoomed in to 5 seconds.
